# Evolutionary histories of coxsackievirus B5 and swine vesicular disease virus reconstructed by phylodynamic and sequence variation analyses

**DOI:** 10.1038/s41598-018-27254-y

**Published:** 2018-06-11

**Authors:** Hui-Wen Huang, Pei-Huan Chu, Chu-Hsiang Pan, Chu-Feng Wang, Chien-Ching Lin, Po-Liang Lu, Yao-Shen Chen, Yong-Ying Shi, Hui-Ju Su, Li-Chiu Chou, Yi-Ying Lin, Hsiao-Fen Lee, Bao-Chen Chen, Tsi-Shu Huang, Yu-Chang Tyan, Chih-Hung Chuang, Yung-Chang Yen, Pei-Yu Chu

**Affiliations:** 1 Department of Anesthesiology, Kaohsiung Chang Gung Memorial Hospital and Chang Gung University College of Medicine, Kaohsiung, 833 Taiwan; 2 Department of Cardiology, Wei-Gong Memorial Hospital, Miaoli, 351 Taiwan; 3 Council of Agriculture, Animal Health Research Institute, New Taipei City, 251 Taiwan; 4 Department of Laboratory Medicine, Kaohsiung Medical University Hospital, Kaohsiung, 807 Taiwan; 5 Department of Medical Laboratory Science and Biotechnology, College of Health Sciences, Kaohsiung Medical University, Kaohsiung, 807 Taiwan; 6 Department of Laboratory Medicine, Tainan Hospital, Ministry of Health and Welfare, Tainan, 700 Taiwan; 7 School of Medicine, College of Medicine, Kaohsiung Medical University, Kaohsiung, 807 ROC Taiwan; 8 Department of Infectious Diseases, Kaohsiung Veterans General Hospital, Kaohsiung, 813 ROC Taiwan; 9 Department of Internal Medicine, National Yang-Ming Medical University, Taipei, 112 ROC Taiwan; 10 Department of Internal Medicine, Kaohsiung Veterans General Hospital, Kaohsiung, 813 ROC Taiwan; 11 Division of Microbiology, Department of Pathology and Laboratory Medicine, Kaohsiung Veterans General Hospital, Kaohsiung, 813 ROC Taiwan; 12 Center for Infectious Disease and Cancer Research, Kaohsiung Medical University, Kaohsiung, 807 Taiwan; 13 Department of Medical Imaging and Radiological Sciences, Kaohsiung Medical University, Kaohsiung, 807 Taiwan; 14 Department of Ophthalmology, Chi Mei Medical Center, Liou-Ying, Tainan, 736 Taiwan; 15 Department of Nursing, Min Hwei College of Health Care Management, Tainan, 736 Taiwan

## Abstract

Coxsackievirus (CV)-B5 is a common human enterovirus reported worldwide; swine vesicular disease virus (SVDV) is a porcine variant of CV-B5. To clarify the transmission dynamics and molecular basis of host switching between CV-B5 and SVDV, we analysed and compared the VP1 and partial 3D^pol^ gene regions of these two viruses. Spatiotemporal dynamics of viral transmission were estimated using a Bayesian statistical inference framework. The detected selection events were used to analyse the key molecules associated with host switching. Analyses of VP1 sequences revealed six CV-B5 genotypes (A1–A4 and B1–B2) and three SVDV genotypes (I–III). Analyses of partial 3D^pol^ revealed five clusters (A–E). The genotypes evolved sequentially over different periods, albeit with some overlap. The major hub of CV-B5 transmission was in China whereas the major hubs of SVDV transmission were in Italy. Network analysis based on deduced amino acid sequences showed a diverse extension of the VP1 structural protein, whereas most sequences were clustered into two haplotypes in the partial 3D^pol^ region. Residue 178 of VP1 showed four epistatic interactions with residues known to play essential roles in viral host tropism, cell entry, and viral decoating.

## Introduction

Coxsackievirus (CV)-B5 is a small, naked, single positive-stranded RNA virus in the *enterovirus* (*EV*)*-B* species of the Picornaviridae family. Enteroviral infections are highly contagious and are typically disseminated via faecal-oral and respiratory routes. As in EV-B, infection with CV-B5 is typically self-limiting; however, it can cause potentially fatal conditions (e.g., encephalitis, myocarditis, and neonatal sepsis-like disease) and chronic autoimmune diseases (e.g., insulin-dependent diabetes mellitus)^1,2^. Approximately half of all documented CV-B5 infections have been in infants who are highly vulnerable to infections^3,4^. The distinctive pattern of CV-B5 circulation is characterized by a sudden peak in infectious activity every 3–6 years. In intervening years, infections are sporadic but generally occur annually^5,6^. In Taiwan, CV-B5 epidemics occurred in 1995, 2002, 2011, and 2015^3^. Interestingly, heavy precipitation (130–200 mm) is a more important risk factor for EV infection (relative risk, RR = 2.45, 1.59–3.78) than for dengue fever (RR = 1.96, 1.53–2.52)^7^ because naked viruses such as EV are more stable during harsh weather conditions such as heavy rainfall compared to enveloped viruses such as dengue virus. For example, EVs can contaminate groundwater even after being washed out of their dominant areas by heavy rainfall. Although the prevalence of EV peaks in warmer seasons, factors such as global warming and migration resulting from economic or political events such as the Syrian conflict^8^ mandate year-round surveillance.

Swine vesicular disease (SVD) was first described in Italy in 1966. Since then, numerous outbreaks have occurred in Europe and Asia^9–11^. Swine vesicular disease is difficult to detect because, although morbidity varies by strain, symptoms are usually mild. Additionally, SVD infections are usually detected during outbreaks of foot and mouth disease, which is clinically indistinguishable from SVD. In 2015, SVD was deleted from the Office of International Epizootics Listed Diseases^12^. In Taiwan, surveillance data show that SVD infections occurred only sporadically during 1997–2000. The pathogenic cause of SVD is the SVD virus (SVDV), which comprises a single serotype. Due to the sequence homology of CV-B5 and SVDV and their antigenic cross-reactivity, SVDV is now classified as a porcine variant of CV-B5^13^. Although pigs are currently the only known SVDV host, researchers hypothesize that SVDV evolved from a shared ancestor of CV-B5, and then crossed the species barrier from human to pig through adaptation^10,11^. Given that biological sequences (DNA, RNA, and protein) are footprints of evolution, sites of sequence variation between ancestor and recent strains reveal not only the evolutionary history of a virus, but also the sequence conserved patterns thought to have functional value for survival^14,15^. Owing to the close genetic relationship between CV-B5 and SVDV, a comparison of their sequence variation signatures could reveal the key molecular sites of cross-host species transmission.

The VP1 gene of EVs encodes outer capsid proteins that determine host range and receptor binding^16^. The RNA-dependent RNA polymerase (RdRp) is a 3D^pol^ gene product that plays an important hereditary role in RNA viruses not only because mutations result in the loss of proofreading activity, but also because RdRp mediates template switching, which is a well-known recombination mechanism in RNA viruses^17^. Both mutation and recombination contribute to the rapid evolution rate needed to maintain viral adaptability under selective pressure. Gene alignment can reveal the evolutionary history of a virus whereas analysis of amino acid (aa) sequence conservation can reveal not only the constraints on protein structure and function, but also the effects of evolutionary dynamics. Therefore, identifying sequence variations in the VP1 and 3D^pol^ regions can reveal the evolutionary characteristics of CV-B5 and SVDV.

Phylodynamic analysis can elucidate the epidemiological, immunological, and evolutionary interactions of a pathogen^18^, whereas selection detection can further reveal how the direction of evolution is affected by interactions between gene sequence and molecular function. Therefore, we hypothesized that combining phylodynamic and epistatic analyses would yield useful information about viral host barrier crossing and adaptation. To clarify the molecular characteristics of CV-B5 and SVDV and the spatiotemporal characteristics of their evolution, a Bayesian statistical inference framework was used to analyse VP1 and partial 3D^pol^ regions in both viruses. Relationships among genealogies were also depicted by haplotype network analysis, and patterns of variation associated with host range switching were analysed by comparing CV-B5 and SVDV sequences.

## Results

### VP1-based phylogenetic and demographic analyses of CV-B5 and SVDV

**Figure 1:**
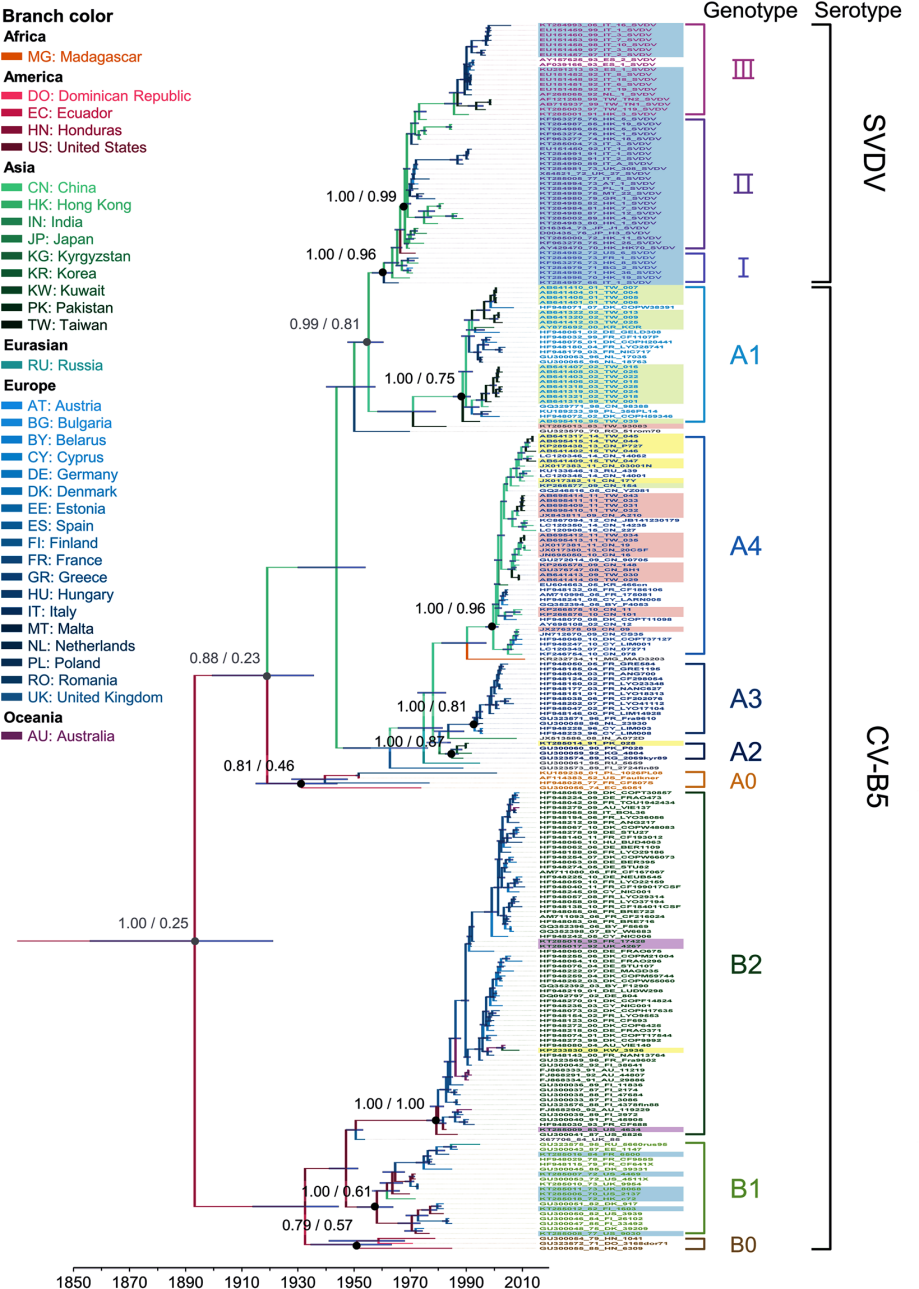
Maximum clade credibility (MCC) phylogeny of 248 VP1 sequences of coxsackievirus B5 and swine vesicular disease virus. For each branch, the colour indicates the most probable location. Blue bars at nodes indicate 95% highest probability density of time to the most recent ancestor. Numbers above major nodes indicate the support value of posterior/location probability. For each strain, the assigned name is indicated on the right, VP1 genotypes are differentiated by text colour, and 3D^pol^ genotypes are differentiated by shade. Branch length is proportional to evolution time, and the scale bar depicts calendar time.

Three datasets were used to estimate spatiotemporal dynamics in the 849-nucleotide (-nt) VP1 region: a CV-B5 dataset (195 sequences isolated from 28 countries during 1952–2015, including 28 Taiwan strains isolated in this study), an SVDV dataset (53 sequences sampled from 14 countries during 1966–2006), and a combined dataset designated 248_data (195 CV-B5 sequences and 53 SVDV sequences). Supplementary Table 1 shows the model compositions used. The maximum clade credibility (MCC) tree and maximum likelihood (ML) tree for 248_data showed that the two main branches evolved from a common ancestor (Fig. 1 and Supplementary Fig. S1). Previous studies showed a bifurcating tree with two major circulating genogroups^19,20^. This study applied the genogroup A and B designations used by Henquell *et al*. because dichotomous grouping is simple and clear^19^. The genotype A strains were prevalent mainly in Asia and Europe, while the genotype B strains were prevalent mainly in America and Europe. The two main branches evolved continuously in separate subclusters. Genotype analysis was limited to sequence with higher than 15% similarity^21^ and nodes with high support values (posterior probability value, PP > 0.9 or bootstrap value >70%), criteria that are widely used to discriminate non-polio enterovirus genotypes^22–26^, including EV-B. Sequential evolution of six CV-B5 genotypes (A1−A4 and B1−B2) were observed in this study. In each genogroup, some ancestor strains were separated from the main subcluster. Compared with the main subcluster, these ancestor strains had a wider range of isolation years, wider distribution of isolation locations, lower sequence similarity, and/or lower nodal support values, and were assigned as 0. For example, genotype A successively yielded A0, A1−A4, and SVDV; and genotype B successively yielded B0, B1, and B2. All SVDV strains were rooted in a single branch of genogroup A and clustered together with genogroup A1. The SVDV was further subdivided into genotypes I−III. Although all 248 strains were rooted together with high support values in both Bayesian Markov chain Monte Carlo (BMCMC) tree and ML trees, these results indicate that SVDV evolved from CV-B5. However, support values on the rooted nodes of SVDV with genogroup A are low (Fig. 1 and Supplementary Fig. S1). Supplementary Figs 2 and 3 also show within-genotype similarities. Dominant sublineages may have circulated for several years before being replaced by newer sublineages with extended branches. Different sublineages may have co-circulated during a given period in the same location, especially in recent decades.

The MCC trees for the CV-B5 and 248_data datasets showed similar topologies and clustering. On rooted branches, both trees had a balanced structure indicating co-survival in bifurcating sub-branches. On terminal branches, both trees had an imbalanced structure indicating temporal and geographic clustering of CV-B5 isolates, especially in those isolated after 2000, with a ladder-like backbone (Fig. 1 and Supplementary Fig. 1). After the prototype strain of CV-B5 appeared in the US in 1952, it reappeared in France and Ecuador in the 1970s and later in Poland in 2001. This cluster, which had a low PP value and low sequence similarity, was not included in the GenBank after 2001. Genotype A1 strains were isolated from Asia (Kyrgyzstan and Pakistan, 1989−1992); whereas genotype A2 strains were isolated from Europe and Asia (1995–2003); genotype A3 strains were isolated from Europe (1996−2005); and genotype A4 strains were isolated from Asia and Europe (2002−2015). In genogroup B, genotype B1 strains had circulated in America and Europe (1970−1985), whereas genotype B2 strains had circulated only in Europe (1983−2011).

Unlike the dichotomous phylogeny of CV-B5, SVDV clustered monophyletically with genotype A1 with high support values. All three genotypes included strains isolated in Europe and Asia but sub-clustered by their isolation locations and timespans. Genotype I comprised strains isolated during 1966−1973, including the prototype strain KT284997 isolated in Italy (1966); Genotype II included strains prevalent during 1972–1992; Genotype ASIII strains were isolated only after 1990. Topology and clustering in the SVDV tree resembled those in the 248_data except for five strains isolated from Hong Kong (1973−1985), which clustered together with genotype III with high support values in 248_data. In contrast, certain earlier Asian strains (isolated from Hong Kong and Japan during 1970–1976) clustered with genotype III in the SVDV tree and had low support values (Fig. 1 and Supplementary Fig. 2).

### VP1-based estimation of time to most recent common ancestor (TMRCA), substitution rates, and viral demographic history

**Figure 2:**
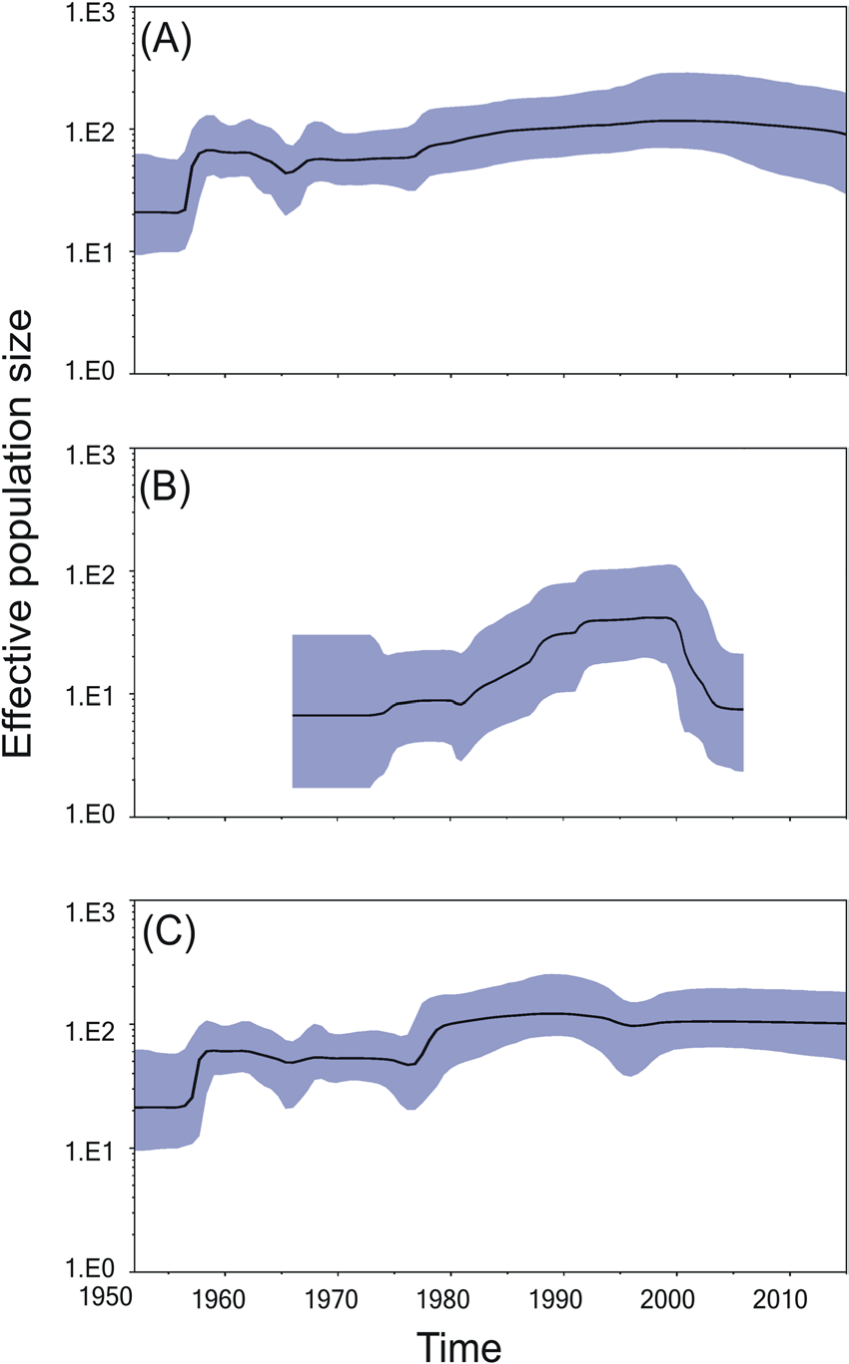
Bayesian skyline plot based on VP1 of (**A**) the 248_data (**B**) swine vesicular disease virus, and (**C**) coxsackievirus B5 sequences. The x-axis shows the time scale in years; the y-axis is the logarithmic effective population size (EPS) scale (Ne, EP; τ, generation time). The thick solid line indicates the median EPS, and the shaded area indicates the 95% highest posterior density.

The analytical results for 248_data resembled those for CV-B5 except that CV-B5 had a slightly higher EPS during 1965–2000. For 248_data, the estimated mean (95% highest probability density, HPD) TMRCAs were 1891 (1856–1920) for CV-B5 and 1955 (1948–1961) for SVDV with an evolution rate of 4.47 × 10^−3^ (3.90 × 10^−3^ –5.05 × 10^−3^) substitutions/site/year (s/s/y). When analysed separately, the TMRCAs were 1895 (1863–1926) for CV-B5 and 1955 (1847–1962) for SVDV, and their evolution rates were 4.76 × 10^−3^ (4.11 × 10^−3^–5.47 × 10^−3^) s/s/y and 3.61 × 10^−3^ (2.87 × 10^−3^–4.34 × 10^−3^) s/s/y, respectively. Figure 2 shows the Bayesian skyline plots (BSPs) of the viral demographic histories for CV-B5, SVDV and 248_data. The results for CV-B5 show that a one-step increase in effective population size (EPS) occurred in 1960. During 1960-2015, the EPS was generally stable, i.e., fluctuation in EPS (e.g., a dip in 1965) did not appreciably affect the 95% credibility interval. On the other hand, the EPS of SVDV started from only 8.8 (1.7–31.1) and gradually increased to a plateau in 1999 before decreasing after 2000.

### 3D^pol^-based phylodynamic analysis

**Figure 3:**
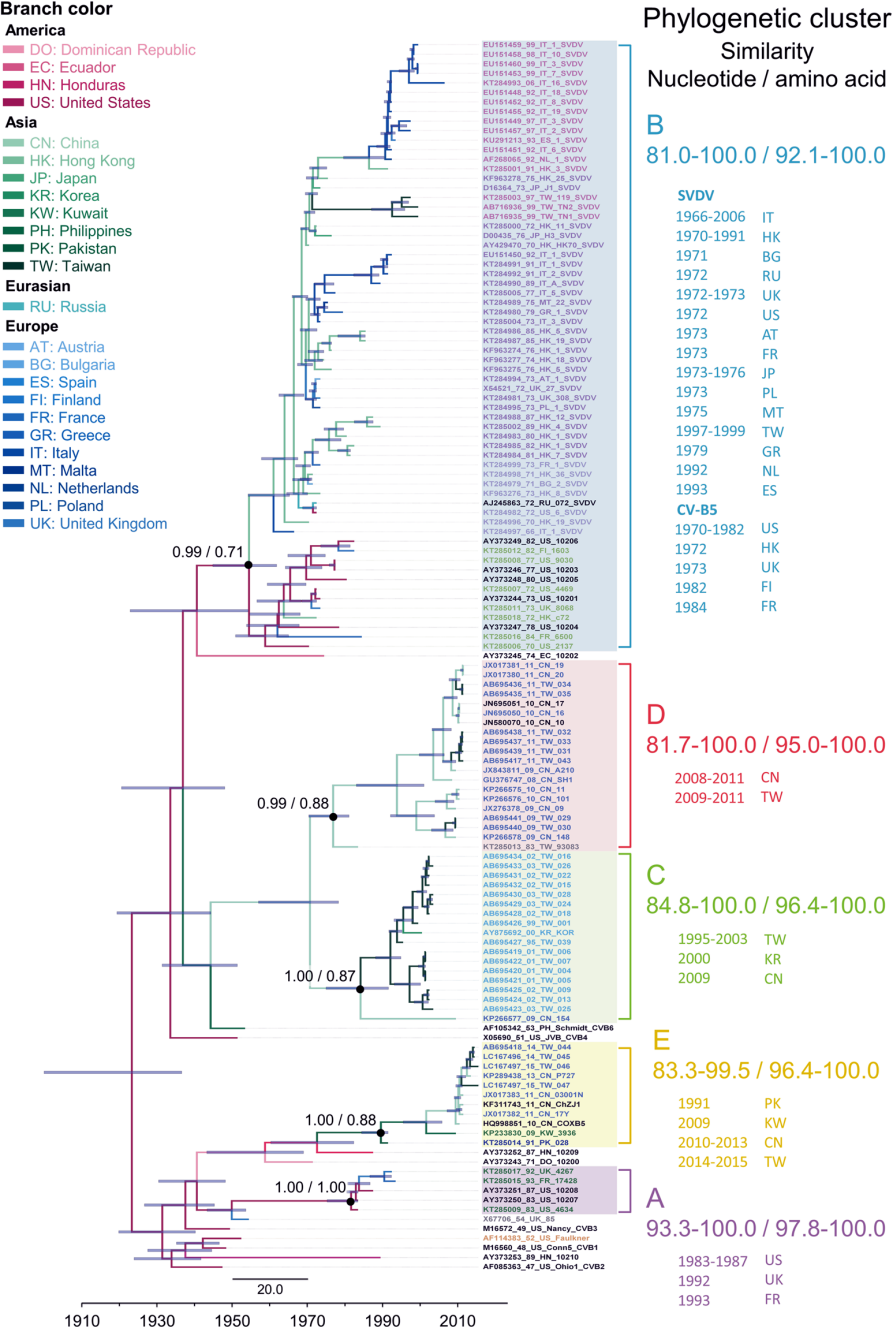
Maximum clade credibility phylogeny of 3D^pol^ sequences of coxsackievirus B5, swine vesicular disease virus, and outgroups. For each branch, the colour indicates the most probable location. Numbers above major nodes indicate the support values. Genotypes and nucleotide/amino acid similarities within genotypes are shown on the right. For each strain, VP1 genotypes are differentiated by colour whereas 3D^pol^ genotypes are differentiated by shade. Branch length is proportional to evolution time, and the scale bar depicts calendar time.

Five phylogenetic clusters (A–E) with high support values were revealed in this partial 3D^pol^-based analysis (Fig. 3). Most prototypical CV-B strains were alone in a separate branch, and most strains were in clusters that corresponded with VP1 genotypes. The cluster A sequences of 3D^pol^ tree included earlier isolates from the United Kingdom, France, and United States, and most of these strains were clustered in genotype B2 of VP1. The cluster B included all SVDV and CV-B5 strains that corresponded with genotype B1 of VP1. The cluster C strains included those isolated from Taiwan during 1995–2003 and one each from Korea (2000) and China (2009), which were mainly clustered in genotype A1 of VP1. The cluster D strains were isolated from Taiwan (1983–2011) and China (2008−2011) and were mainly clustered in genotype A4 of VP1. The cluster E included strains isolated from Dominica (1971), Hong Kong (1987), Pakistan (1991), China (2010–2013), and Taiwan (2013–2015); these were also clustered in genotype A4 of VP1. This partial 3D^pol^-based analysis-revealed a notable incongruence: SVDV clustered together with genotype B1 in the 3D^pol^ tree instead of with genotype A1 in the VP1 tree.

### Geographic transmission of CV-B5 and SVDV

**Figure 4:**
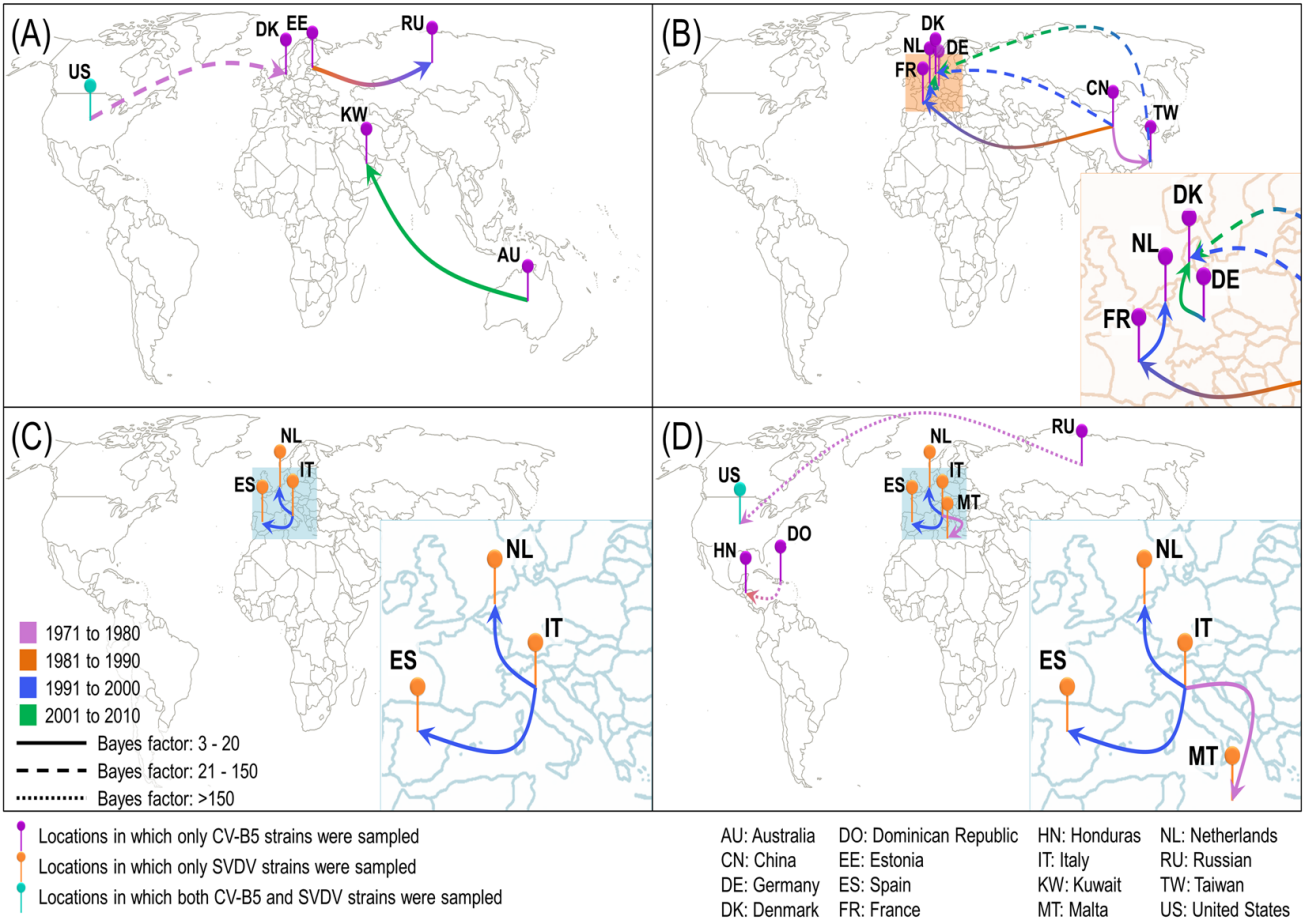
Major routes of non-zero geographic dispersal. Routes were determined for (**A**) the VP1 region based on the CV-B5 dataset, (**B**) the VP1 region based on the SVDV dataset, (**C**) the VP1 region based on 248_data, and (**D**) the 3D^pol^ region in CV-B5, SVDV, and outgroups. Lines between different locations indicate transmission routes with high support values (BF >3) and are coloured according to transmission duration. Arrows indicate the transmission direction. Pushpins indicate sampling locations and are coloured by virus sampling. Country abbreviations are shown below. The map is available online at https://commons.wikimedia.org/wiki/File:World_map_blank_black_lines_4500px_monochrome.png. This map was released under a Creative Commons Attribution-Share Alike 3.0 International license at https://creativecommons.org/licenses/by-sa/3.0/deed.en.

The diffusion histories for both viruses were reconstructed using Bayesian estimation and annotated using the Spatial Phylogenetic Reconstruction of Evolutionary Dynamics 3 (SpreaD3) program. Based on the analysis of VP1, 11 transmission routes were supported by a Bayes factor >3 (Fig. 4). The 248_data revealed three transmission routes: one from the United States (to Denmark), one from Estonia (to Russia), and one from Australia (to Kuwait). The CV-B5 dataset revealed six transmission routes: one from Germany (to Denmark), three from China (to Taiwan, France, and Denmark), one from France (to the Netherlands), and one from Taiwan (to Denmark). The SVDV dataset revealed two routes: both from Italy (to the Netherlands and Spain). The analysis of the 3D^pol^ dataset revealed five transmission routes supported by a BF >3: one from Dominica (to Honduras), one from Russia (to the United States), and three from Italy (to the Netherlands, Spain, and Malta). In the 3D^pol^ dataset, Italy, the Netherlands, Spain, Malta, and Russia only had SVDV strains, suggesting that these transmission routes were important in SVDV transmission. Notably, transmissions from Italy to Netherlands and from Italy to Spain were supported by both analyses based on VP1 region of SVDV data and 3D^pol^ data, revealing the important role played by Italy in SVDV transmission.

### Detection of variation in VP1 and 3D^pol^ regions

Although no recombination events were detected in either the VP1 (structural) or partial-3D^pol^ (nonstructural) regions, the incongruity of constructed phylogenies indicated that recombination occurred between these regions. To investigate the codon sequence composition of SVDV, we analysed 27 CV-B5 and 50 SVDV full codon sequences in GenBank. According to the SimPlot results (Supplementary Fig. 3), genotype A1 of CV-B5 and genotype B1 of CV-B5 had high similarity in the VP1 and 3D^pol^ regions, respectively. Additionally, the similarity between CV-B5 and SVDV was lower (<75%) within nt 2650–3000. The segmental arrangement of various CV-B5 genotypes in SVDV indicated that multiple recombination events have occurred as SVDV evolved.

**Figure 5:**
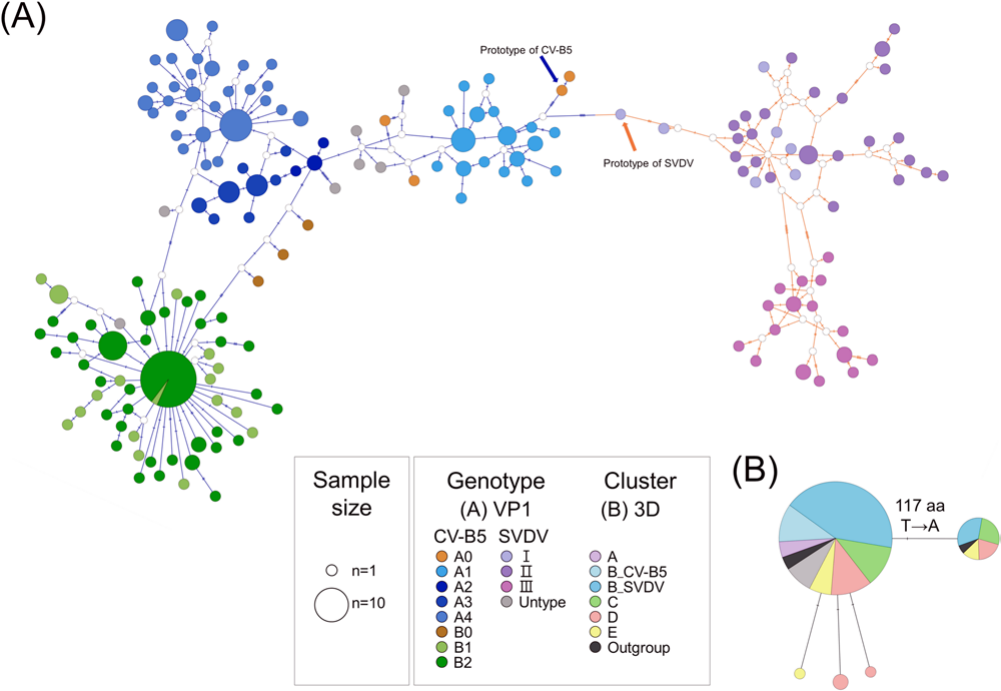
Graphic depiction of haplotype networks in the (**A**) VP1 and (**B**) partial 3D^pol^ regions of CV-B5 and SVDV at the amino acid sequence level. Circle size is proportional to haplotype frequency. Genotypes are distinguished by colour. Pie charts depict each haplotype across multiple genotypes. Small hollow circles represent unsampled intermediate sequences. Numbers of mutations are indicated by stripes on connecting branches.

The genealogical relationships of deduced aa sequences in the VP1 and 3D^pol^ regions of CV-B5 and SVDV were further visualized using the network in population analysis with reticulate trees (PopART) program^27^. Figure 5A clearly shows that based on the analysis of the VP1 region, CV-B5 and SVDV strains clearly clustered in separate branches (blue branch in the left and salmon branch in the right). The prototype strain of CV-B5 and SVDV were located near the junction of the blue branch and salmon branch. Both CV-B5 and SVDV networks revealed radical extensions, which were roughly grouped by genotype. The network revealed that the branch separated into genotype A1 and SVDV from prototype strain of CV-B5, each extending towards opposite directions. Next, the CV-B5 branch continuously extended A1 and formed a network that consist of genotypes A2 to A4 (on the upper-left of Fig. 5A), and further extends to the network consisting of strains formed by mixing of genotypes B1 and B2 (in the lower-left of Fig. 5A). Notably, this genogroup B network revealed a major group (the biggest circle) with short random extensions by mixing of genotype B1 and B2 strains (Supplementary Fig. S4). The biggest haplotype mixing by random extension short branches suggests that the founder effect was established by the fittest haplotype.

The prototype SVDV strain, from the junction of blue and salmon branches, extended to a mixed network of genotype I and II strains (light and dark purple circle on the right). This network then prolonged into two genotype II branches (upper-right) and genotype III network (lower-right), which corroborated the results of phylogenetic analysis and indicated that the SVDV strains had evolved with a spatiotemporal trend (Supplementary Fig. 5).

In the 3D^pol^ region, all strains were clustered into one large and small haplotype cluster each (Fig. 5B). The small haplotype included the prototype CV-B1 strain, 12 CV-B5 strains (four cluster C, six cluster D, and two cluster E), and five SVDV strains (Supplementary Fig. 6). All other strains were grouped together in the large haplotype cluster and its short branches. The presence of numerous haplotype clusters with short random extension branches also indicated the existence of a common ancestor and establishment of dominant traits.

**Figure 6:**
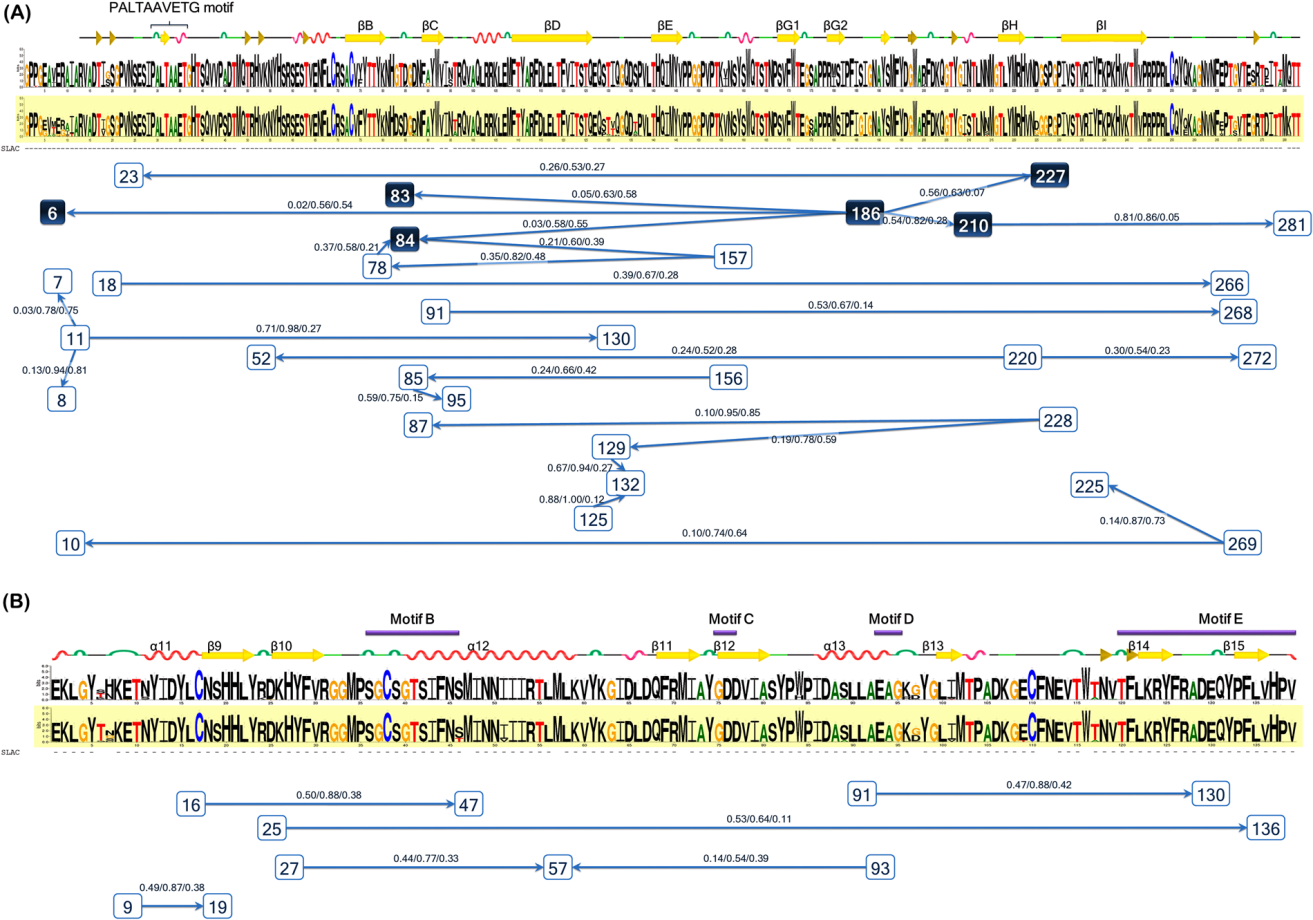
Graphic depiction of sequence variations in the (**A**) VP1 and (**B**) partial 3D^pol^ regions. The CV-B5 consensus sequence is shown on the upper line, and the SVDV sequence is shown on the lower and is shadowed. The consensus sequence graphs were generated by the WebLogo program. The secondary structure guide is located at the top of consensus sequence graph (PDB ID code 1MQT for VP1 of SVDV and 3CDW for 3D^pol^ of CV-B3). Dashes under the consensus sequence graph indicate negative selection sites, identified by SLAC method. Epistatic interactions are shown at the bottom of the figure. Each square represents a residue position that participated in at least one interaction with a marginal posterior probability (PP) exceeding a default cutoff of 0.5. Sites with multiple epistastic interactions are highlighted with a dark blue box. Arrows between squares indicate the epistastic direction between residues. The PP values are presented in the following order: PP(→)/PP(↔)/PP(←). Epistatic interactions are identified by BGM. Both SLAC and BGM were implemented on the DataMonkey website.

Patterns conserved between CV-B5 and SVDV were visualized using the WebLogo program^28^. The two viruses showed aa sequence similarity in almost every VP1 and 3D^pol^ residue (Fig. 6). The single likelihood ancestor counting (SLAC) method obtained d*N*/d*S* values of 0.0334 and 0.0265 for the VP1 and 3D^pol^ regions, respectively. A d*N*/d*S* value of <1 indicates negative selection, in which the aa sequence tends to be fixed. Neither VP1 nor 3D^pol^ revealed sites of significant positive selection by SLAC. Both regions showed scattered sites of significant negative selection. However, the two viruses had five conserved nonsynonymous mutation sites (G83D, T84S, S186G, N210S, and S227G) in VP1. The epistatic analysis based on 248_data revealed 25 pairs of epistatic interactions (PP >0.5). Intriguingly, position 186 showed multiple epistatic interactions with positions 83, 84, 210, 227, and 6. These epistatic interactions occurred among all five nonsynonymous mutation sites. The partial 3D^pol^ region revealed no positive sites and no mutation pattern common to CV-B5 and SVDV. Six pairs of epistatic interactions were identified; however, none of them differed between CV-B5 and SVDV. The only residue difference between these two haplotypes was residue 117 immediately before motif E, where T in the large haplotype cluster was replaced by A in the small haplotype cluster.

## Discussion

For a historical reconstruction of the spatiotemporal transmission of CV-B5 and SVDV, sequence changes during host switching were analysed in 834 nt of the VP1 region (248 sequences, 248_data) and in 420 nt of a partial 3D^pol^ region (129 sequences, including prototypical sequences of CV-B1–B4 and CVB6 as an outgroup). Phylodynamic analyses were used to estimate the epidemic, evolutionary, and immunological characteristics of CV-B5 and SVDV transmission. Analyses of evolutionary history revealed haplotype networks with deduced aa sequences. However, virus phylogenetic analysis should be carefully considered owing to several limitations. Ancestral sequences are undersampled owing to insufficient data deposited in GenBank, whereas recently isolated strains are oversampled owing to the use of improved laboratory techniques. Meanwhile, virulent strains are reported more frequently than viruses with low pathogenicity. Furthermore, the lack of reports of a virus in a given country does not mean that the virus is not currently circulating in that country. Thus, the results of the analysis should be cautiously interpreted in these conditions.

Recombination occurs frequently in EV-B, particularly in the non-structural region^29,30^. Previous results obtained from the phylogenetic analyses of structural and nonstructural regions of EV-B are often incongruent^31,32^. Earlier studies have reported that clusters in the structural region (e.g., VP1) are monophyletic by serotype whereas clusters in the nonstructural region (e.g., 3D^pol^) are monophyletic by species (e.g., EV-B). This study did not directly detect recombination events within the VP1 or 3D^pol^ regions. However, CV-B5, similar to other serotypes in EV-B species, showed incongruent phylogenetic results for the VP1 and 3D^pol^ regions. The VP1 tree had a monophylogenetic topology, but the 3D^pol^ region was rooted together with other serotypes in EV-B^23^. The incongruent phylogenies constructed from structural (i.e., VP1) and nonstructural (i.e., 3D^pol^) regions indicate the occurrence of recombination events between these two regions. On the countrary, the SVDV phylogenetic trees constructed in this study were also incongruent; SVDV strains clustered together with genogroup A of CV-B5 in the VP1 tree, but clustered together with genogroup B of CV-B5 in the 3D^pol^ tree. Interestingly, all SVDV isolates were monophyletic not only in the VP1 tree, but also in the 3D^pol^ tree, and support values were high in both regions. This result supports a previous hypothesis that SVDV originated from a common ancestor after the host was transferred from human to pig^11^, as the 3D^pol^ tree of SVDV clustered together with SVDV strains alone instead of rooting at the species level with other EV-B strains.

A previous report has suggested Hong Kong as an important transmission hub^10^. Hong Kong is a small region with limited natural resources, and is consequently reliant on imports. Thus, transmission of swine-associated pathogens to Hong Kong via live pigs or pork has been reported^33^. However, no transmission route from Hong Kong has been detected with high BF value in the current study. The geographic analysis revealed that China played important roles in CV-B5 transmission, and Italy played important roles in SVDV transmission.

Owing to a high selectivity for immune escape, VP1 has the lowest similarity of all EV genes. A “75/85% rule” is the common scheme used for molecular typing of EV species. That is, complete or partial VP1 sequences are assumed to have the same serotype if their nt or aa sequence similarity exceeds 75%^34^. A cutoff value of 15% sequence similarity is widely used in EV genotype discrimination^21^. Previous CV-B5 phylogenetic analyses have reported 2–5 genotypes, and all of these studies revealed a similar topology^19,20,35,36^. In the current study, the TMRCAs for two genogroups, both of which included SVDV strains, were similar to those reported by Gullberg, et. al. (Supplementary Table. 3)^27^. Continuous bipartite branches have been reported in genotype A^19^. This topology indicates that the sublineages co-circulated in a wide geographic area during their evolution. The genogroup B consists of one imbalanced ladder-like backbone and strains that mainly circulated in Europe^19^. We observed that ladder-like backbones were common not only in genogroup B but also in the terminal branches of recently circulating genotypes such as genotype A1 and genotype A4. These two genotypes included the strains after 2000. Improved laboratory techniques have reduced the loss of branch data. Given that tree imbalance is mainly determined by directional selection^18^, these data imply that, after evolution of the fittest sequence, the population size increased, but variation was fixed. That is, balanced structures suggest a founder effect in adaptive haplotypes. Thus, haplotype size and a mix of early and recent strains are also useful fitness indicators.

Sequence comparisons showed that VP1 starts with a conserved PALTAVETGHT motif (associated with viral uncoating) followed by a conical β-barrel commonly seen in the viral capsid protein. This β-barrel is composed of eight antiparallel β-strands (strand B to strand I). The main variations among VP1 are in the N- and C-terminus and in the loops that connect the β strands, especially in the outermost BC and DE loops^37^. As reported in previous studies, five conserved mutations (83, 84, 201, 227 and 186) of VP1 were identified in CV-B5 and SVDV^19,38^. A novel finding of the current study is the multiple epistatic interactions observed in the 186 residue. Positions 83 and 84 are located in antigenic site I (within the BC-loop), position 210 is located in the putative site of coxsackievirus and adenovirus receptor (CAR) binding, position 186 is located in the hydrophobic pocket, position 227 is located within the five-fold axis, and position 6 is located in the N-terminus before the PALTAVETGHT motif^19^. Notably, CAR is a cellular receptor of CV-B, and mutations of residues 83 and 84 are often reported in association with neutralization escape. The hydrophobic pocket is a determinant of pocket factor selectivity and, like the cellular receptor, is an important determinant of viral tropism in Picornaviridae. Meanwhile, pocket factor expulsion is associated with capsid destabilization. Subsequent extrusion of the VP1 N termini (positions 5 to 18) then forms the viral genome propeller tip^26^. Thus, all positions involved in the multiepistatic interaction were associated with viral tropism and viral decoating.

The RdRp proteins were organized as a closed right hand structure with three domains (fingers, palm, and thumb) consisting of seven motifs in the order G, F, A–E from the N terminus to the C terminus^39,40^. In the partial 3D^pol^ region, this study compared aa residues in positions 254–392 of motifs B to E in the palm region. Motif B is involved in the specific selection of rNTPs as substrates during RdRp polymerization. Motif C includes the strongly conserved GDD motif and has a catalytic residue Asp329 between β11 and β12. Motif E (β14 and β15) is the transition region between the palm and thumb domains^34^. The comparisons showed no signature variation between CV-B5 and SVDV, which indicated that this partial 3D^pol^ region does not functionally differ between human and swine hosts.

In conclusion, comparing homologous virus variants in different host species can reveal genetic components needed for viral infection and adaptation to a different host. The VP1 gene has had a particularly important role in host tropism and transmission through the host barrier whereas the partial 3D^pol^ region is associated with viral heredity and thus does not differ in a host switch from human to pig. This study used sequencing data to reconstruct the spatiotemporal transmission histories of CV-B5 and SVDV and detected selection events to reveal the key molecular sites associated with different host species.

## Methods

### Specimen collection and ethics statement

For each year of positive CV-B5 isolation (1999–2015), 28 isolates were randomly selected from a pool of de-identified virus stock from two medical centres in southern Taiwan (Kaohsiung Veterans General Hospital and Kaohsiung Medical University Hospital). The ethics committees of both hospitals approved this study. Experiments were limited to viral isolates obtained from clinically necessary laboratory procedures; medical histories were not reviewed, and no patients were harmed. As such, informed consent was not required. The SVDV sequences were obtained from the Animal Health Research Institute, Taiwan (AHRI-TW). Samples used in this study were obtained from certified farms that voluntarily sent samples to AHRI-TW. Animals were investigated according to the regulations established by the Central Epidemic Command Center of Taiwan. As such, this study did not require approval by the ethics committee of the institutional review board.

### Viral RNA extraction, reverse transcriptase (RT-)PCR, and sequencing

Sampled viral strains were amplified in confluent rhabdomyosarcoma cells. The procedures for RNA purification, RT-PCR, and sequencing were performed as described previously^41,42^. Briefly, viral RNA was extracted from 200 μl of viral culture supernatant using the QIAmp viral RNA purification kit (Qiagen, Chatsworth, CA, USA). The One-Step RT-PCR kit (Qiagen) was used to perform RT-PCR with 200 ng total RNA using specific primer pairs (Table 1) for 35 amplification cycles. Purified PCR products were sequenced with the ABI Prism Ready Reaction Dideoxy Terminator cycle sequencing kit (Model 3730 v.3.4; Applied Biosystems, Foster City, CA, USA). The sequences for forward and reverse strands were obtained simultaneously and edited with Sequence Navigator v.3.01 software (Applied Biosystems). The obtained VP1 (880 nt) and partial 3D^pol^ (420 nt) gene sequences were submitted to GenBank under the accession numbers listed in Supplementary Table 2.

**Table 1:** Primer sets used for amplification and sequencing of Coxsackievirus B5 (CV-B5) and swine vesicular disease virus (SVDV).

Target virus	Target site-Primer name	Sequence	Reference
CV-B5	VP1-2400(F)	5′-GCTTTGTGTCTGCMTGYAATGA-3′	CDC-TW
CV-B5	VP1-222R(R)	5′-CICCIGGIGGIAYRWACAT-3′	^42^
CV-B5	VP1-292(F)	5′-MIGCIGYIGARACNGG-3′	^42^
CV-B5	VP1-011(R)	5′-GCICCIGAYTGITGICCRAA-3′	^42^
SVDV	VP1-1(F)	5′-ACGATTTYTCAGTTAGGATGCTCAAGG-3′	AHRI-TW
SVDV	VP1-1(R)	5′-CCAACGTACACRGCACCAGA-3′	AHRI-TW
SVDV	VP1-2(F)	5′-CTCAACTGCGYCGGAAGCTC-3′	AHRI-TW
SVDV	VP1-2(R)	5′-CATACATTATTTGGTGGGTGAGCAC-3′	AHRI-TW
Both	3D-PY/CC/1(F)	5′-GTAGCAATGAGGCAGACATTTGG-3′	This study
Both	3D-PY/CC/2(R)	5′-AGGATCTTTAGTCCACCTAATGGATTCG-3′	This study
Both	3D-PY/03(F)	5′-GTYACMTATGTGAARGATG-3′	^41^
Both	3D-PY/04(R)	5′-CTTCATTGGCATTACTGGATG-3′	^41^

F: forward; R: reverse; CDC-TW, Centers for Disease Control, Taiwan; AHRI-TW, Animal Health Research Institute, Taiwan.

### Detection of sequence variation and selection

Full-length VP1 and partial 3D^pol^ (positions 6671–7090 of accession no. AF114383) sequences of CV-B5 and SVDV were obtained using the Basic Local Alignment Search Tool program. Multiple sequence alignments were performed with T-coffee^43^. Sequences were manually excluded if they (1) lacked isolation date or location data or (2) if they had ambiguously codons, nonsense, or frameshift mutations. Sequences with the same isolation location and year were then stratified by random sampling. Recombination events were detected using Recombination Detection Program v.3.44 with default settings^44^ and SimPlot v.3.5.1^12^. Pairwise comparisons of nt and aa sequences were estimated with the p-distance method in the MEGA v.7 program^45^. The SLAC method was used to estimate site-specific selection, i.e., the ratio of non-synonymous substitutions to synonymous substitutions (d*N*/d*S*). A *p*-value less than 0.05 was considered statistically significant. The Spidermonkey/Bayesian graphical model (BGM) was used to detect sites with epistatic interaction, and PP <0.5 was considered statistically significant. All programs used to detect selection, including SLAC and BGM, were implemented on the DataMonkey website^46^. For a comprehensive comparison of population differences between CV-B5 and SVDV, variations in deduced aa sequences between VP1 and 3D^pol^ proteins of both viruses were graphically visualized with the WebLogo^24^. The genealogical relationships were further visualized by using the method developed by Templeton, Crandall, and Singh (TSC)^47^ to construct a network in PopART program^27^.

### Phylogenetic and phylodynamic analysis

The best-fit substitution model by jMODELTEST v.2.1.7 program^48^ under Akaike information criteria was performed. Maximum likelihood phylogenetic analyses were conducted using the MEGA v.7 program. Further, the Bayesian evolutionary analysis sampling trees (BEAST) v.1.8.4 program was used for BMCMC tree analysis^49^. All sampled sequences were stamped with isolation times and locations. Eight candidate model compositions (substitution-clock-tree) were used for Bayesian estimation in all four datasets (three for VP1 sequences and one for partial 3D sequences). All model compositions included the SRD06 substitution model, relaxed uncorrelated clock model with either exponential distribution or lognormal distribution, and one of the four coalescent tree priors: constant size, exponential growth, logistic growth, and BSP (1*2*4 = 8). The best-fit model composition for each dataset was selected with the path sampling and stepping-stone methods. The MCMC tree analysis was also used to co-estimate rate of growth, substitution rate, and TMRCA. The TRACER v.1.6 program was used to calculate effective sample size (ESS) based on the stationarity of post-burn-in distributions and estimated parameters, which were considered significant if ESS >200. These estimated parameters were expressed as mean value with 95% HPD. FigTree v.1.4.3 software was used to visualize the MCC tree. Nodal support was estimated by calculating PP, and significant support was defined as PP > 0.9. A BEAST log file with rate indicators was generated by asymmetric Bayesian stochastic search variable selection in location reconstruction. The SPREAD3 v0.9.6 program was used to convert the tree into KML format and to estimate non-zero expectancy rates^50^. Discrete sampling locations were plotted as the centre of the country in which the strain was isolated.

## Electronic supplementary material

Supplementary Information
