# Supplementary material for: Evolutionary histories of coxsackievirus B5 and swine vesicular disease virus reconstructed by phylodynamic and sequence variation analyses
Source: Sci Rep. 2018 Jun 11;8:8821. doi: 10.1038/s41598-018-27254-y (PMC5995886; doi:10.1038/s41598-018-27254-y)
Supplement: Supplementary file 1 — Supplementary Information [file 41598_2018_27254_MOESM1_ESM.pdf]

Scientific Reports

Supplementary information

Evolutionary histories of coxsackievirus B5 and swine vesicular disease virus reconstructed by phylodynamic and sequence variation analyses

Hui-Wen Huang, Pei-Huan Chu, Chu-Hsiang Pan, Chu-Feng Wang, Chien-Ching Lin, Po-Liang Lu, Yao-Shen Chen, Yong-Ying Shi, Hui-Ju Su, Li-Chiu Chou, Yi-Ying Lin, Hsiao-Fen Lee, Bao-Chen Chen, Tsi-Shu Huang, Yu-Chang Tyan, Chih-Hung Chuang, Yung-Chang Yen*, and Pei-Yu Chu*

Content

Supplementary Tables 3

Supplementary Table 1. Best models according to path sampling (PS) and stepping-stone (SS) methods 3

Supplementary Table 2. Accession numbers and isolation years for coxsackievirus B5 isolates used in this work. 5

Supplementary Table 3. Estimated time to most recent common ancestor (TMRCA) for coxsackievirus B5 (CV-B5) and swine vesicular disease virus (SVDV) 6

Supplementary Figure 7

Supplementary Figure S1. Molecular phylogenetic analysis of 248 VP1 sequences by maximum likelihood method. 7

Supplementary Figure S2. Maximum clade credibility phylogeny of 195 VP1 sequences of coxsackievirus B5. 8

Supplementary Figure S3. Maximum clade credibility phylogeny of 53 VP1 sequences of swine vesicular diseases virus. 9

Supplementary Figure S4. Comparison of complete codon composition between swine vesicular disease virus (SVDV) and each genotype of coxsackievirus B5 (CV-B5). 10

Supplementary Figure S5. Detail and strain list of haplotype clusters in network analysis based on VP1 11

Supplementary Figure S6. Detail and strain list of haplotype clusters in network analysis based on partial $3D^{pol}$ 12

Supplementary Tables

Supplementary Table 1. Best models according to path sampling (PS) and stepping-stone (SS) methods

(A) Comparison of model compositions used to analyze 248 VP1 sequences in coxasackievirus B5 (CV-B5) and swine vesicular disease virus (SVDV)

Marginal likelihood				
Model composition	SS	PS	SS−PS	Best_SS−Current_SS
UCED CON	-22423.672	-22423.926	0.254	2422.567
UCED EXP	-21288.623	-21288.284	-0.339	1287.519
UCED BSP	-22295.941	-22295.425	-0.516	2294.837
UCLD CON	-20004.306	-20004.308	0.002	3.202
**UCLD EXP**	**-20001.105**	**-20001.183**	**0.079**	**0.000**
UCLD BSP	-20585.757	-20586.125	0.367	584.653

(B) Comparison of model compositions used to analyze 195 VP1 sequences in CV-B5

Marginal likelihood				
Model composition	SS	PS	SS−PS	Best_SS−Current_SS
UCED CON	-19081.700	-19081.271	-0.430	10305.969
UCED EXP	-19078.877	-19078.235	-0.642	10303.146
UCED BSP	-9299.281	-9297.658	-1.623	523.550
UCLD CON	-19033.999	-19034.358	0.358	10258.268
**UCLD BSP**	**-8775.731**	**-8775.770**	**0.039**	**0.000**

(C) Comparison of model compositions used to analyze 53 VP1 sequences in SVDV

Marginal likelihood				
Model composition	SS	PS	SS−PS	Best_SS−Current_SS
UCED CON	-4873.548	-4873.593	0.0455	5.5357
UCED EXP	-4876.441	-4876.475	0.0349	8.4284
UCED LOG	-4987.528	-4987.103	-0.426	119.516
UCED BSP	-4878.649	-4877.871	-0.778	10.637
**UCLD CON**	**-4868.012**	**-4867.817**	**-0.196**	**0.000**
UCLD EXP	-4874.165	-4874.278	0.114	6.152
UCLD BSP	-4869.841	-4869.937	0.096	1.829

(D) Comparison of model compositions used to analyze the 3D^{pol} region

Marginal likelihood<br>Model composition	SS	PS	SS−PS	Best_SS−Current_SS
UCED CON	-7608.728	-7608.321	-0.407	10.638
UCED EXP	-7605.131	-7605.019	-0.112	7.041
**UCED BSP**	**-7598.090**	**-7598.286**	**0.196**	**0.000**
UCLD CON	-7612.752	-7612.781	0.029	14.662
UCLD EXP	-7611.946	-7612.118	0.172	13.856
UCLD BSP	-7599.238	-7599.524	0.286	1.148

- The best performing model is shown in bold font. The highest marginal likelihood results indicate the best fit and are shown in bold font.
- Each model was composed of a substitution model, a clock model, and a tree model. The candidate model compositions included one substitution model (SRD06), one of the two clock models (relaxed uncorrelated clock with exponential distribution (UCED) or relaxed uncorrelated clock with lognormal distribution (UCLD)) , and one of the four tree prior models (constant size (CON), exponential growth (EXP), logistic growth (LOG), or Bayesian skyline plot (BSP)).
- Consequently, eight different candidate model compositions were used for each dataset. Only major estimated parameters with effective sample sizes >200 are shown. For all analyses, PS and SS were performed using 100 steps, and a chain length of 10,000,000, and sampling was performed after every 1000 chain length.

Supplementary Table 2. Accession numbers and isolation years for coxsackievirus B5 isolates used in this work.

Strain name	Isolation year	Accession no. of VP1 region	Accession no. of $3D^{pol}$ region
001	1999	AB641316	AB695426
004	2001	AB641404	AB695420
005	2001	AB641405	AB695421
006	2001	AB641401	AB695419
007	2001	AB641410	AB695422
009	2002	AB641320	AB695425
013	2002	AB641322	AB695424
015	2002	AB641406	AB695432
016	2002	AB641407	AB695434
018	2002	AB641321	AB695428
022	2002	AB641403	AB695431
024	2003	AB641319	AB695429
025	2003	AB641412	AB695423
026	2003	AB641408	AB695433
028	2003	AB641318	AB695430
029	2009	AB641414	AB695441
030	2009	AB641413	AB695440
031	2011	AB695409	AB695439
032	2011	AB695410	AB695438
033	2011	AB695411	AB695437
034	2011	AB695412	AB695436
035	2011	AB695413	AB695435
039	1995	AB695416	AB695427
043	2011	AB695414	AB695417
044	2014	AB695415	AB695418
045	2014	AB641317	LC167496
046	2015	AB641402	LC167497
047	2015	AB641409	LC167498

Supplementary Table 3. Estimated time to most recent common ancestor (TMRCA) for coxsackievirus B5 (CV-B5) and swine vesicular disease virus (SVDV)

	Gullberg, et. al.; 2010	Henquell, et. al.; 2013	Bruhn et. al. 2015	Current study
Target codon	VP1	VP1	VP3-VP1	VP1
Sequences include SVDV	+	-	+	+
Best fit model	SRD06-UCLD-BSP	SRD06-UCLD-BSP	SRD06-UCLD-BSP	SRD06-UCLD-EXP
TMRCA				
CV-B5	1854 (1807-1898)			1891 (1856-1920)
Genogroup	II: 1913 (1887-1935)	A: 1908 (1849-1948)		A: 1919 (1899-1935)
	I: 1933 (1916-1947)	B: 1941 (1912-1962)		B: 1933 (1914-1945)
SVDV			1961 (1954-1965)	1955 (1848-1961)

- Estimated TMRCA values express as means (95% highest probability density).
- SRD06: Shapiro-Rambaut-Drummond-2006, UCLD: relaxed uncorrelated clock with lognormal distribution, EXP: exponential growth, BSP: Bayesian skyline plot.

Supplementary Figure

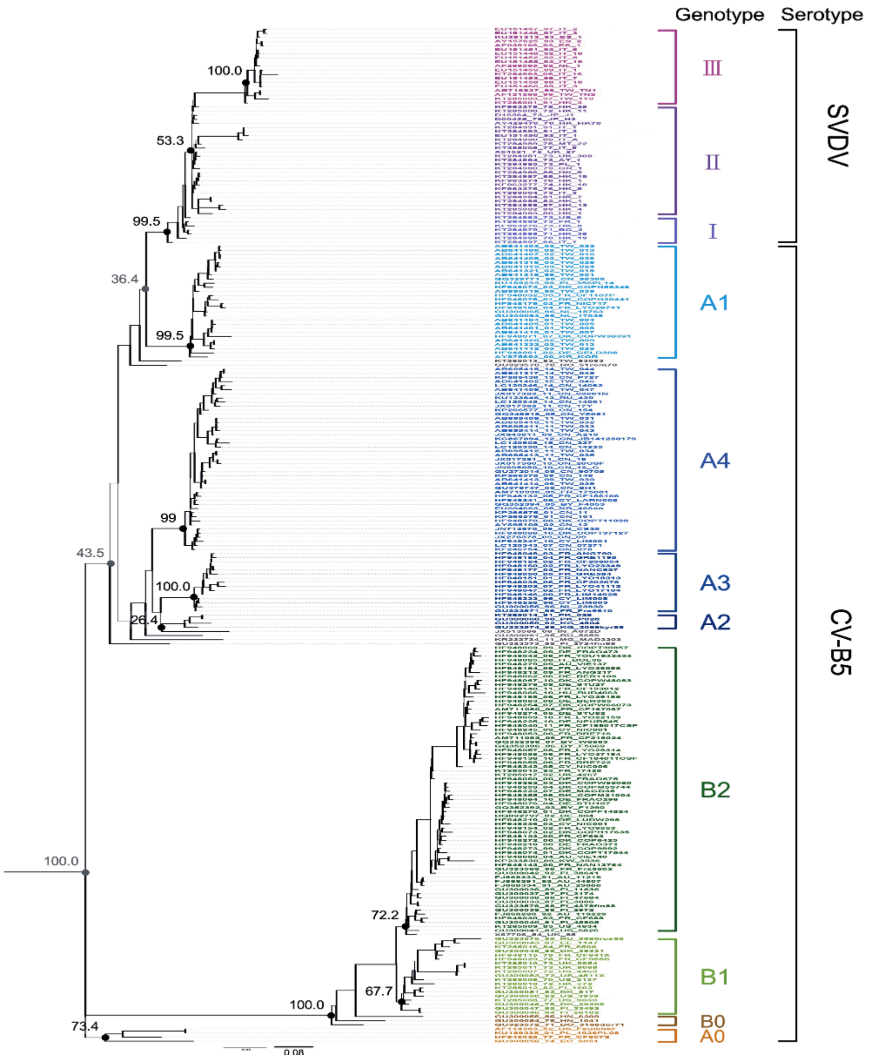

Supplementary Figure S1. Molecular phylogenetic analysis of 248 VP1 sequences by maximum likelihood method. According to Akaike information criteria, the general time reversible model with discrete gamma distribution and invariance was selected as the best-fit substitution model by the jMODELTEST v.2.1.7 program. Initial tree for heuristic search were obtained automatically by applying neighbor-join and BioNJ algorithms to a matrix of pairwise distances estimated using the maximum composite likelihood approach, followed by selecting the topology with superior log likelihood value. A discrete gamma distribution (+*G*, parameter = 0.6682) with four categories was used to model evolutionary rate differences among sites. The rate variation model allowed some sites to be evolutionarily invariable ([+*I*], 43.2038% sites). Evolutionary analyses were conducted using MEGA7. Although all 248 strains rooted together with high support value, the support values at nodes of diversion of SVDV from genogroup A or genotype A1 are low. Results indicated that the SVDV strains evolved from a common ancestor, but doesn't support SVDV evolved from genogroup A or genotype A1.

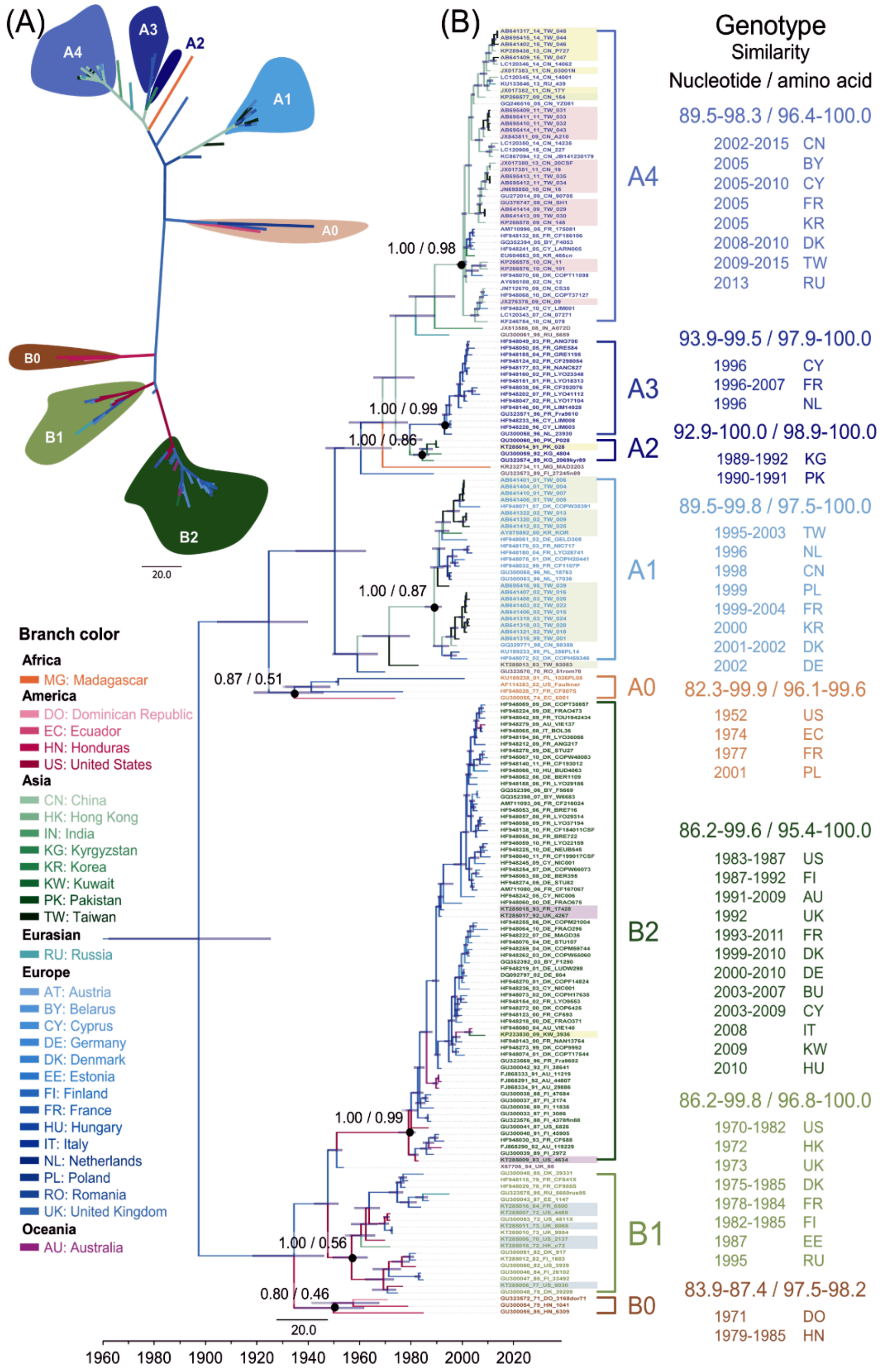

Supplementary Figure S2. Maximum clade credibility phylogeny of 195 VP1 sequences of coxsackievirus B5. (A) Unrooted tree. Each shadow represents different genotype. (B) Rooted tree. For each genotype within-genotype nucleotide/amino acid similarities and isolation locations/years shows in the right side. Data show as those in Fig. 1.

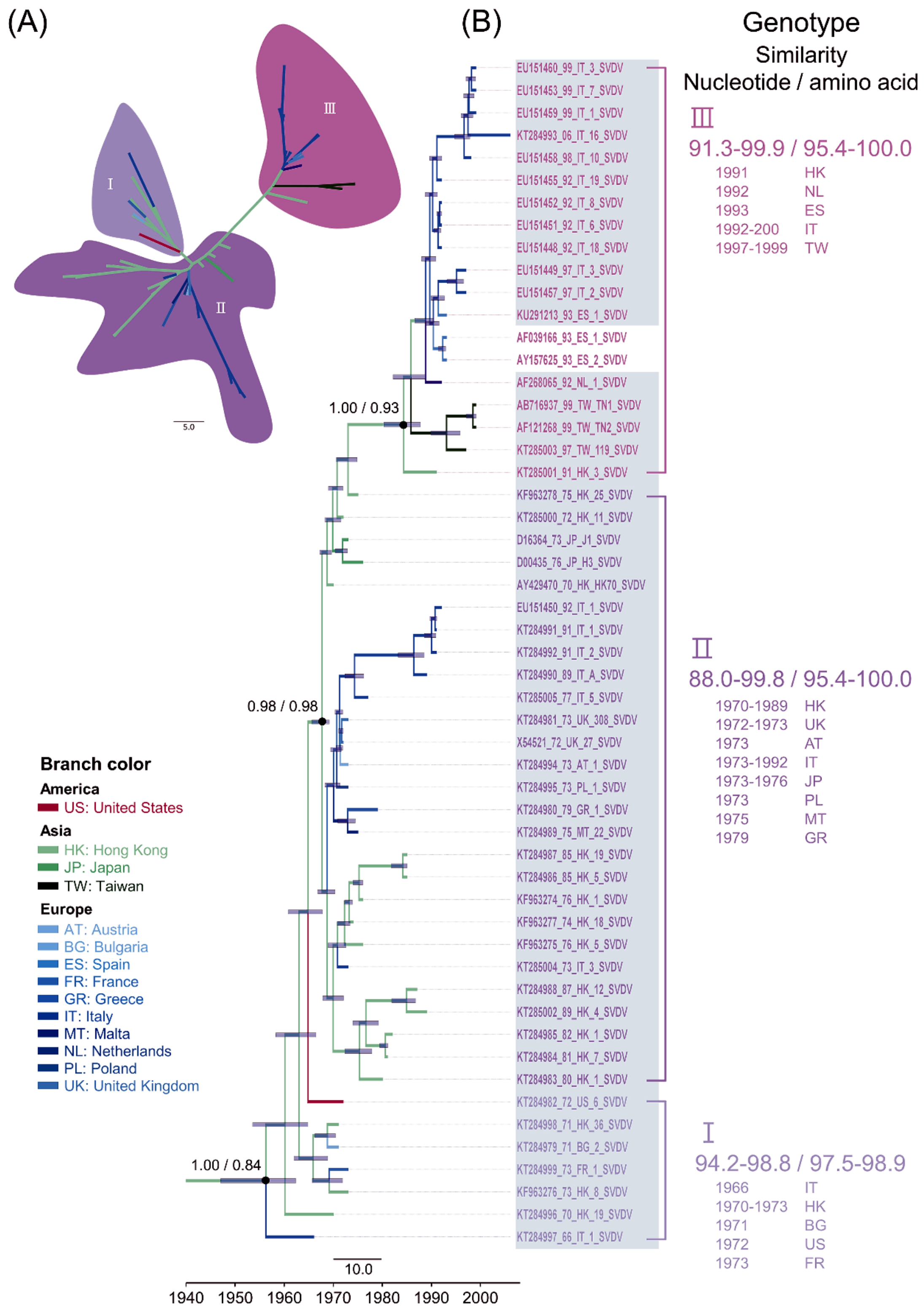

Supplementary Figure S3. Maximum clade credibility phylogeny of 53 VP1 sequences of swine vesicular diseases virus. (A) Unrooted tree. (B) Rooted tree. Data are identical to those in Figure 1 except for within-genotype nt/aa similarities and isolation locations/years (right side of figure).

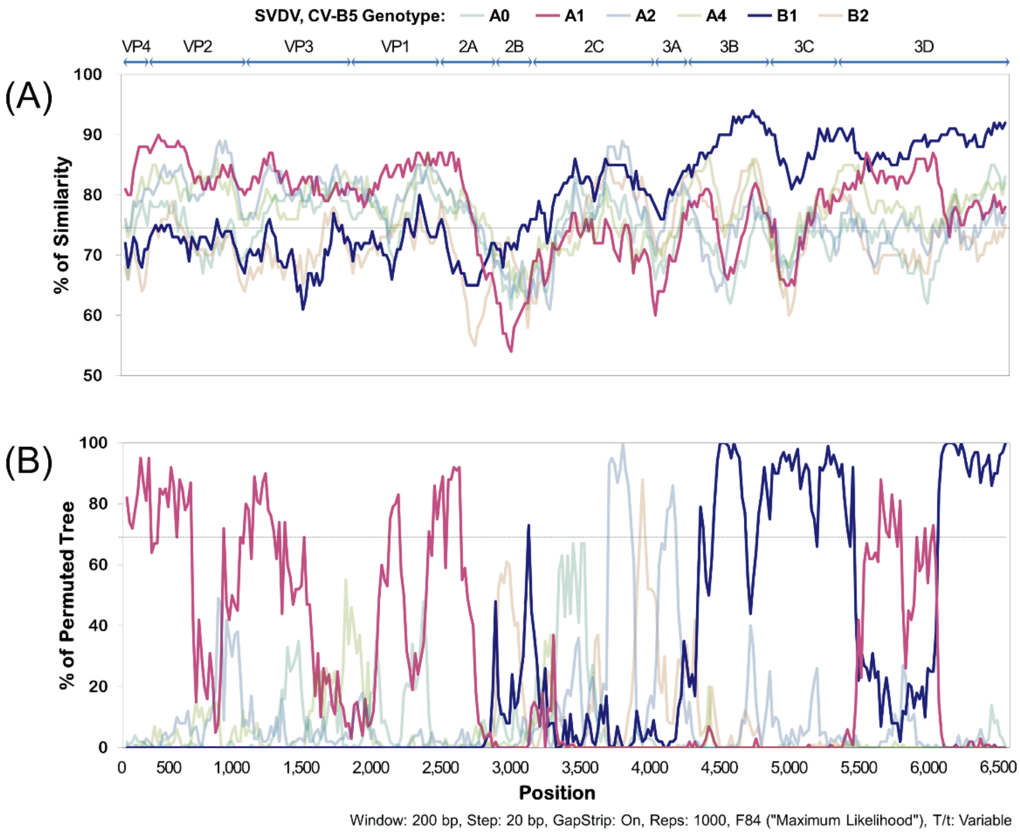

Supplementary Figure S4. Comparison of complete codon composition between swine vesicular disease virus (SVDV) and each genotype of coxsackievirus B5 (CV-B5). The SimPlot program was used to analyze 77 complete codons in CV-B5 and SVDV sequences sampled from GenBank. No genotype A3 or genotype B0 were available for CV-B5. The maximum likelihood model was used with an empirically determined transition/transversion ratio. The window width was set to 200 nucleotide (nt) with a step size of 20 nt. The blue bar at the top indicates the gene region. (A) Plot of similarity over nt position. Percentage of similarity is shown on the y axis. The dashed line indicates a cutoff of 75% in the similarity. (B) Plot of permutation over nt position. Percentage of permutation is shown on the y axis. The dashed line indicates a recombination cutoff of 70% in the permutation test.

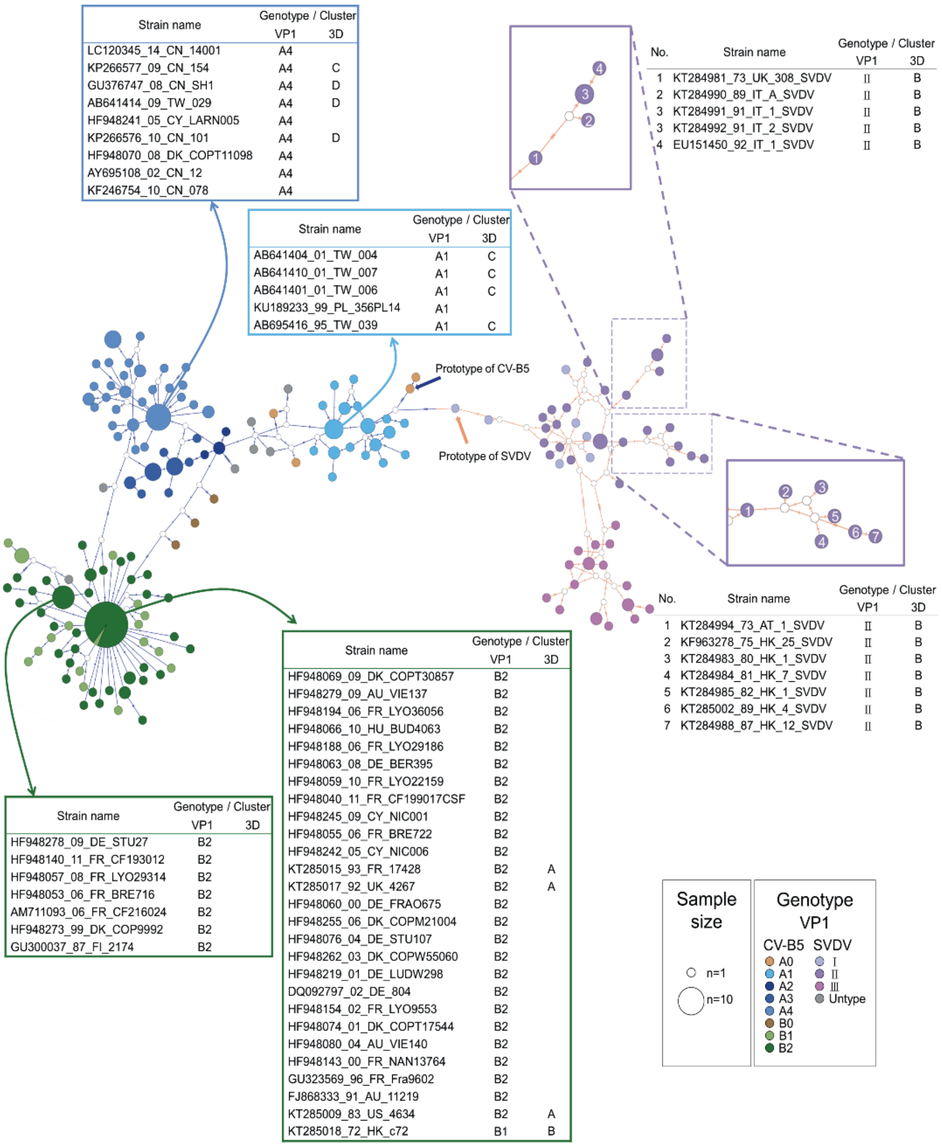

Supplementary Figure S5. Detail and strain list of haplotype clusters in network analysis based on VP1

Strain name	Genotype / Cluster VP1	3D
EU151459_99_IT_1_SVDV	III	B
EU151458_98_IT_10_SVDV	III	B
EU151460_99_IT_3_SVDV	III	B
EU151453_99_IT_7_SVDV	III	B
KT284993_06_IT_16_SVDV	III	B
EU151448_92_IT_18_SVDV	III	B
EU151452_92_IT_8_SVDV	III	B
EU151455_92_IT_19_SVDV	III	B
EU151449_97_IT_3_SVDV	III	B
EU151457_97_IT_2_SVDV	III	B
KU291213_93_ES_1_SVDV	III	B
EU151451_92_IT_6_SVDV	III	B
AF268065_92_NL_1_SVDV	III	B
KT285001_91_HK_3_SVDV	III	B
D16364_73_JP_J1_SVDV	II	B
KT285003_97_TW_119_SVDV	III	B
AB716936_99_TW_TN2_SVDV	III	B
AB716935_99_TW_TN1_SVDV	III	B
KT285000_72_HK_11_SVDV	II	B
D00435_76_JP_H3_SVDV	II	B
AY429470_70_HK_HK70_SVDV	II	B
EU151450_92_IT_1_SVDV	II	B
KT284991_91_IT_1_SVDV	II	B
KT284992_91_IT_2_SVDV	II	B
KT284990_89_IT_A_SVDV	II	B
KT285005_77_IT_5_SVDV	II	B
KT284989_75_MT_22_SVDV	II	B
KT284980_79_GR_1_SVDV	II	B
KT285004_73_IT_3_SVDV	II	B
KT284986_85_HK_5_SVDV	II	B
KT284987_85_HK_19_SVDV	II	B
KF963274_76_HK_1_SVDV	II	B
KF963277_74_HK_18_SVDV	II	B
KF963275_76_HK_5_SVDV	II	B
KT284994_73_AT_1_SVDV	II	B
X54521_72_UK_27_SVDV	II	B
KT284981_73_UK_308_SVDV	II	B
KT284995_73_PL_1_SVDV	II	B
KT284985_82_HK_1_SVDV	II	B
KT284984_81_HK_7_SVDV	II	B
KT284999_73_FR_1_SVDV	I	B
KT284979_71_BG_2_SVDV	I	B
KF963276_73_HK_8_SVDV	I	B
AJ245863_72_RU_072_SVDV		B
KT284982_72_US_6_SVDV	I	B
KT284996_70_HK_19_SVDV	I	B
KT284997_66_IT_1_SVDV	I	B
AJ245863_72_RU_072_SVDV		B
KT284982_72_US_6_SVDV	I	B

Strain name	Genotype / Cluster VP1	3D
KT285017_92_UK_4267_G	B2	A
KT285015_93_FR_17428_G	B2	A
AY373251_87_US_10208		A
AY373250_83_US_10207		A
KT285009_83_US_4634_G	B2	A

Strain name	Genotype / Cluster VP1	3D
AY373249_82_US_10206		B
KT285012_82_FI_1603	B1	B
KT285008_77_US_9030	B1	B
AY373246_77_US_10203		B
AY373248_80_US_10205		B
KT285007_72_US_4469	B1	B
AY373244_73_US_10201		B
KT285011_73_UK_8068	B1	B
KT285018_72_HK_c72	B1	B
AY373247_78_US_10204		B
KT285016_84_FR_6500	B1	B
KT285006_70_US_2137	B1	B

Strain name	Genotype / Cluster VP1	3D
AB695434_02_TW_016	A1	C
AB695433_03_TW_026	A1	C
AB695431_02_TW_022	A1	C
AB695432_02_TW_015	A1	C
AB695430_03_TW_028	A1	C
AB695429_03_TW_024	A1	C
AB695428_02_TW_018	A1	C
AB695426_99_TW_001	A1	C
AY875692_00_KR_KOR	A1	C
AB695427_95_TW_039	A1	C
AB695425_02_TW_009	A1	C
AB695424_02_TW_013	A1	C
AB695423_03_TW_025	A1	C
KP266577_09_CN_154	A4	C

Strain name	Genotype / Cluster VP1	3D
JX017381_11_CN_19	A4	D
JX017380_11_CN_20	A4	D
AB695436_11_TW_034	A4	D
AB695435_11_TW_035	A4	D
JN695051_10_CN_17		D
JN695050_10_CN_16	A4	D
JN580070_10_CN_10		D
AB695438_11_TW_032	A4	D
AB695437_11_TW_033	A4	D
AB695439_11_TW_031	A4	D
AB695417_11_TW_043	A4	D
JX843811_09_CN_A210	A4	D
KP266575_10_CN_11	A4	D
KP266576_10_CN_101	A4	D
JX276378_09_CN_09	A4	D
KT285013_83_TW_93083		D

Strain name	Genotype / Cluster VP1	3D
AB695418_14_TW_044	A4	E
LC167496_14_TW_045	A4	E
JX017383_11_CN_03001N_G	A4	E
KF311743_11_CN_ChZJ1_G	A4	E
JX017382_11_CN_17Y_G	A4	E
HQ998851_10_CN_COXB5	A4	E
KP233830_09_KW_3936_G	B2	E
KT285014_91_PK_028_G	A2	E

Strain name	Genotype / Cluster VP1	3D
AF085363_47_US_Ohio1_CVB2		
M16572_49_US_Nancy_CVB3		
X05690_51_US_JVB_CVB4		
AF105342_53_PH_Schmidt_CVB6		
AF114383_52_US_Faulkner	A0	
AY373253_89_HN_10210		
X67706_54_UK_85		
AY373252_87_HN_10209		
AY373243_71_DO_10200		
AY373245_74_EC_10202		

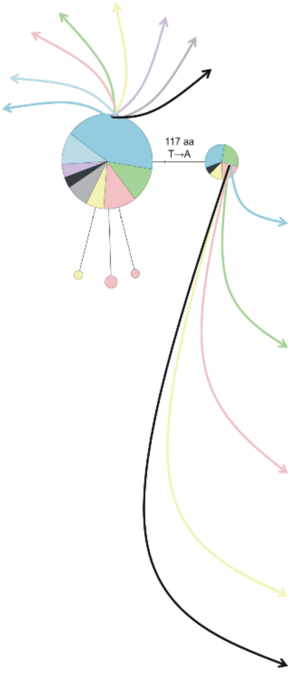

Strain name	Genotype / Cluster VP1	3D
KF963278_75_HK_25_SVDV	II	B
KT284988_87_HK_12_SVDV	II	B
KT285002_89_HK_4_SVDV	II	B
KT284983_80_HK_1_SVDV	II	B
KT284998_71_HK_36_SVDV	I	B

Strain name	Genotype / Cluster VP1	3D
AB695419_01_TW_006	A1	C
AB695422_01_TW_007	A1	C
AB695420_01_TW_004	A1	C
AB695421_01_TW_005	A1	C

Strain name	Genotype / Cluster VP1	3D
AB695441_09_TW_029	A4	D
AB695440_09_TW_030	A4	D
KP266578_09_CN_148	A4	D
AB695441_09_TW_029	A4	D
AB695440_09_TW_030	A4	D
KP266578_09_CN_148	A4	D

Strain name	Genotype / Cluster VP1	3D
LC167497_15_TW_046	A4	E
KP289438_13_CN_P727	A4	E

Strain name	Genotype / Cluster VP1	3D
M16560_48_US_Conn5_CVB1		

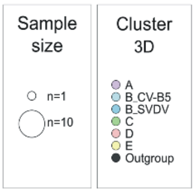

Supplementary Figure S6. Detail and strain list of haplotype clusters in network analysis based on partial $3D^{pol}$
